# Peroxiredoxin III Protects Tumor Suppressor PTEN from Oxidation by 15-Hydroperoxy-eicosatetraenoic Acid

**DOI:** 10.1155/2019/2828493

**Published:** 2019-09-15

**Authors:** Ying Zhang, Jiyoung Park, Seong-Jeong Han, Yongwoon Lim, Iha Park, Jong-Suk Kim, Hyun Ae Woo, Seung-Rock Lee

**Affiliations:** ^1^Department of Biochemistry, Department of Biomedical Sciences, Research Center for Aging and Geriatrics, Research Institute of Medical Sciences, Chonnam National University Medical School, Gwangju 501-190, Republic of Korea; ^2^College of Pharmacy, Graduate School of Pharmaceutical Sciences, Ewha Womans University, Seoul 120-750, Republic of Korea; ^3^COTDE Inc., 19-3, Ugakgol-gil, Susin-myeon, Cheonan-si, Chungcheongnam-do 330-882, Republic of Korea; ^4^Department of Biochemistry, Institute of Medical Science, Chonbuk National University Medical School, Jeonju 560-182, Republic of Korea

## Abstract

Phosphatase and tensin homolog deleted on chromosome 10 (PTEN) is a lipid and protein phosphatase that coordinates various cellular processes. Its activity is regulated by the reversible oxidation of an active-site cysteine residue by H_2_O_2_ and thioredoxin. However, the potential role of lipid peroxides in the redox regulation of PTEN remains obscure. To evaluate this, 15-hydroperoxy-eicosatetraenoic acid (15s-HpETE), a lipid peroxide, was employed to investigate its effect on PTEN using molecular and cellular-based assays. Exposure to 15s-HpETE resulted in the oxidation of recombinant PTEN. Reversible oxidation of PTEN was also observed in mouse embryonic fibroblast (MEF) cells treated with a 15s-HpETE and Lipofectamine mixture. The oxidative dimerization of thioredoxin was found simultaneously. In addition, the absence of peroxiredoxin III aggravated 15s-HpETE-induced PTEN oxidation in MEF cells. Our study provides novel insight into the mechanism linking lipid peroxidation to the etiology of tumorigenesis.

## 1. Introduction

Lipoxygenases (LOX) are a heterogeneous family of enzymes that catalyze the insertion of molecular oxygen into polyunsaturated fatty acids (PUFAs), such as arachidonic acid (AA) and linoleic acid (LA), into the corresponding hydroperoxyl derivates, which can be potent inflammatory and prooxidant mediators [1, 2]. 15-Lipoxygenase (15-LOX), a member of the LOX family, is widely expressed in different organisms [3–8]. 15-LOX metabolizes AA to form 15(s)-hydroperoxyeicosatetraenoic acid (15s-HpETE), the oxidative precursor of 15-hydroxyeicosatetraenoic acid (15s-HETE). 15s-HpETE, 15s-HETE, and many of their analogous metabolites have important physiological functions. However, the end-products of lipid peroxidation have demonstrated mutagenicity [2, 9], providing further evidence that inflammation plays an important role in carcinogenesis via its ability to increase cellular oxidative stress. Increased levels of lipid peroxides have also been linked to the pathogenesis of a variety of human diseases through cellular oxidative damage, including neurodegeneration, atherosclerosis, type II diabetes, metabolic disorders, solid tumors, and hematologic malignancies [3, 10–12]. The redox status is also altered in cancer cells, which may result from increased levels of lipid peroxides [12]. Emerging evidence has suggested that 15s-HpETE-induced membrane lipid peroxidation and free radical generation [9] may exert proinflammatory properties and contribute to endothelial cell injury [13]. Both 15s-HpETE and 15s-HETE were shown to inhibit the growth of cultured human chronic myelogenous leukemia K562 cells by a mechanism associated with reactive oxygen species (ROS) [11, 14, 15]. 15s-HpETE and 15s-HETE formed during inflammation have divergent effects on angiogenesis [16]. 15s-HETE promoted pulmonary artery inflammation by activating the NF-*κ*B pathway [17]. Intradermal injection of 15s-HpETE induced inflammatory symptoms, such as plasma exudation, in rabbits [18]. Although 15s-HpETE has been implicated in the pathogenesis of multiple chronic diseases, its specific molecular target remains unclear.

Phosphatase and tensin homolog deleted on chromosome 10 (PTEN) is a member of the protein tyrosine phosphatase (PTP) superfamily. It is a potent tumor suppressor gene, frequently lost in a variety of human sporadic cancers [19], diabetes, and inherited syndromes, such as Cowden disease and Lhermitte-Duclos syndrome [20]. The primary cellular substrate of PTEN is phosphatidylinositol-3,4,5-triphosphate (PIP3), a lipid second messenger molecule generated by the action of phosphatidylinositol-3-kinase (PI3K). PI3Ks can be activated by a series of stimuli, including insulin, cytokines, neurotransmitters, peptide growth factors, and hormones [21–24]. PIP3 activates serine-threonine kinase protein kinase B (PKB/Akt) and 3-phosphoinositide-dependent kinase (PDK) [25]. By hydrolyzing PIP3, PTEN blocks PI3K signaling activities, such as membrane recruitment and the activation of Akt, thereby inhibiting cell proliferation, growth, survival, and metabolism [26–28]. Functional loss of PTEN results in cancer susceptibility and favors tumor progression. PTEN is negatively regulated through oxidation of its active-site cysteine. Numerous studies have demonstrated that the catalytic activity of PTEN was modulated by ROS, resulting in its catalytic inhibition [29, 30]. H_2_O_2_-oxidized PTEN forms a disulfide bond between cysteine residues Cys^124^ and Cys^71^. Oxidized PTEN can be reversibly converted back to its reduced form by intracellular-reducing systems, such as the thioredoxin (Trx) and glutaredoxin (Grx) systems [30–32]. Trx is a highly conserved antioxidant protein, which comprises the Trx system with selenoprotein thioredoxin reductase (TrxR) and NADPH. Trx maintains the thiol-related redox balance status and plays a pivotal role in the regulation of redox signaling [33, 34]. Oxidative stress-mediated dimerization of Trx can delay the reduction of its active-site disulfide by TrxR, resulting in inactivation of the Trx system [35].

We previously reported that exogenous organic peroxides and hydroperoxides can cause the irreversible oxidation of PTEN by impairing the cellular Trx system [34, 36]. The ability of lipid peroxides to oxidize PTPs has been reported previously [37], as well as the PTEN oxidation by unidentified arachidonic acid metabolites [38]. As a member of the PTP superfamily, PTEN may be a preferential molecular target of lipid peroxides. Thus, the objective of this study was to investigate the effect of lipid peroxide on the redox state of PTEN using an endogenous lipid peroxide 15s-HpETE as a model.

## 2. Materials and Methods

### 2.1. Materials and Reagents

Recombinant wild-type PTEN was purified as described previously [30]. 15s-HpETE and 15s-HETE were purchased from Cayman Chemical (Ann Arbor, MI, USA). NAP-5 Sephadex G25 columns were purchased from GE Healthcare Life Sciences (Little Chalfont, UK). Dulbecco's modified Eagle's medium (DMEM), fetal bovine serum (FBS), Lipofectamine 2000 transfection reagent, and anti-actin antibody were purchased from Thermo Fisher Scientific (Waltham, MA, USA). The PTEN and Trx antibodies were prepared as described previously [39, 40]. Anti-rabbit IgG horseradish peroxidase-conjugated antibody was purchased from Ab Frontier (Daejeon, Korea).

### 2.2. Cell Culture and Treatment

Mouse embryonic fibroblasts (MEFs) were prepared at embryonic day 13.5 from embryos obtained by mating Prdx III^+/−^ mice. All cells were cultured in DMEM supplemented with 10% FBS and maintained in a humidified 5% CO_2_ incubator at 37°C. The cells were seeded into 6-well plates and cultured in complete medium supplemented with 10% FBS to reach 80% confluence. After rinsing three times with phosphate-buffered saline (PBS), the cells were maintained in FBS-free DMEM for 30 min. Lipofectamine 2000 transfection reagent was used to assist lipid peroxide transfer across the cell membranes. After 10 *μ*M 15s-HpETE was mixed with Lipofectamine 2000 transfection reagent, the mixture was added to the culture plate, followed by incubation for the indicated times. The reactions were stopped by removal of the culture medium. The cells were washed three times with cold PBS.

### 2.3. Oxidation of Recombinant PTEN

Recombinant PTEN was oxidized during the course of purification. It was prereduced with 1 mM DTT for 2 h and then passed through a NAP-5 Sephadex G25 column preequilibrated with PTEN assay buffer (100 mM Tris-HCl (pH 8.0), 2 mM EDTA, and 0.1% BSA) to remove DTT before treatment with 15s-HpETE. PTEN assay buffer was deoxygenated under a stream of argon gas before use. Prereduced PTEN was exposed to 5 *μ*M 15s-HpETE for the indicated times or to varying concentrations for 30 min at room temperature. The reactions were terminated with 2 mM NEM. NEM was used to prevent artificial redox reactions by blocking the thiol groups.

### 2.4. Analysis of Redox Status of PTEN by Immunoblotting

Oxidative modifications of PTEN specifically involved in the formation of intramolecular disulfide bonds were readily identified by nonreducing sodium dodecyl sulfate polyacrylamide gel electrophoresis (SDS-PAGE) and immunoblot analysis, as described previously [39]. After incubation, cells were washed three times with ice-cold PBS and lysed with NP-40 lysis buffer (20 mM Tris-HCl (pH 7.5), 150 mM NaCl, 5% glycerol, 0.1% NP-40, 1 mM phenylmethylsulfonylfluoride, and protease inhibitor cocktail) containing 10 mM NEM. Protein concentrations were measured with the BCA protein assay kit (Thermo Fisher Scientific). The samples were subjected to nonreducing electrophoresis gel-loading buffer (60 mM Tris (pH 6.8), 25% glycerol, 2% SDS, and 0.5% bromophenol) or reducing gel-loading buffer, followed by immunoblotting for PTEN.

## 3. Results

### 3.1. *15*s*-HpETE-Induced Oxidation of Recombinant PTEN*

**Figure 1:**
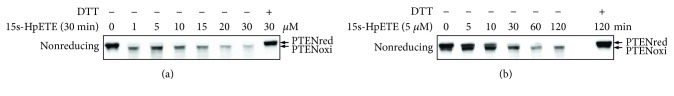
Effects of 15s-HpETE on the redox state of recombinant PTEN. Recombinant PTEN was prereduced with 1 mM DTT for 120 min, followed by passing through a NAP-5 column to remove excess DTT and exposure to various concentrations of 15s-HpETE for 30 min (a) or 5 *μ*M 15s-HpETE for the indicated times (b). The reaction was quenched by 2 mM NEM to block free thiols for 10 min. The samples were then treated with or without 1 mM DTT for reduction and subjected to nonreducing SDS-PAGE, followed by immunoblotting for PTEN.

To investigate the effects of 15s-HpETE on PTEN, purified recombinant PTEN was first assayed. Prereduced recombinant PTEN was incubated with increasing concentrations of 15s-HpETE for 30 min (Figure 1(a)) or increasing periods of time with 5 *μ*M 15s-HpETE (Figure 1(b)) at room temperature. After incubation, NEM was added to block free sulfhydryls to quench further reactions. After exposure of recombinant PTEN to 15s-HpETE, the faster migrating bands in nonreducing SDS-PAGE were increased compared to those in the absence of 15s-HpETE. We previously demonstrated that the faster migrating bands correspond to oxidized PTEN [30]. However, the appearance of PTEN was decreased by 15s-HpETE treatment in a concentration-dependent manner (Figure 1(a)). The loss of immunoblot signal might be due to the loss of sticky protein during oxidation or conformational changes of the PTEN molecule. The addition of DTT to oxidized PTEN (far right lane) resulted in a nearly complete recovery of immunoblot band intensity, suggesting that conformational alteration occurred. PTEN can form homodimers at the plasma membrane and in the solution [41, 42]. Homodimerized PTEN is in an active conformation and exerts lipid phosphatase capability on PIP3 [41]. 15s-HpETE-induced PTEN oxidation was increased with increasing incubation times (Figure 1(b)). These observed results showed that PTEN was oxidized by 15s-HpETE and that the thiol group might be involved in the oxidation because the PTEN oxidized by 15s-HpETE was reduced by DTT.

### 3.2. Reversible Oxidation of PTEN by 15s-HpETE in MEF Cells

**Figure 2:**
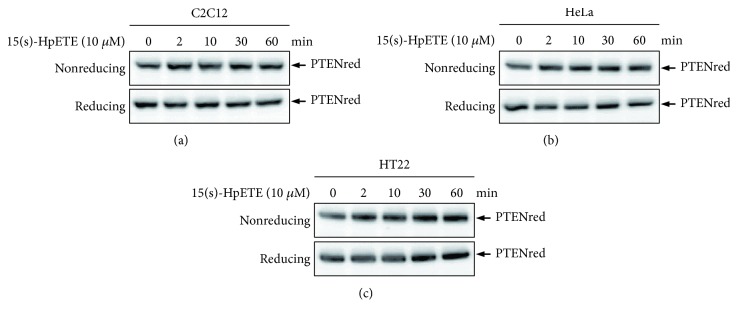
Effects of 15s-HpETE on the redox state of PTEN in C2C12, HeLa, and HT22 cells. The cells were treated with 10 *μ*M 15s-HpETE for the indicated times. Cellular protein extracts were then alkylated with 10 mM NEM and subjected to nonreducing or reducing SDS-PAGE, followed by Western blot analysis using antibodies to PTEN.

To substantiate the findings obtained from recombinant protein, various cell lines, including C2C12, HeLa, and HT22 cells, were further treated with 15s-HpETE to analyze whether endogenous cellular PTEN was an oxidation target. Confluent monolayers of cells were rinsed with PBS and incubated in serum-free growth medium containing 10 *μ*M 15s-HpETE for increasing periods of time (0, 2, 10, 30, and 60 min). The redox status of PTEN was then monitored. Surprisingly, 15s-HpETE treatment was unable to induce PTEN oxidation in the tested cell lines (Figure 2). This was presumably due to the inability of 15s-HpETE to penetrate the cell membranes [9] because of the negative charge imparted by its carboxylate group and the phospholipid-like structure of the cell membrane. Even if a small amount of 15s-HpETE entered the cells, it might be scavenged and degraded rapidly by cellular antioxidants before exerting its effect.

**Figure 3:**
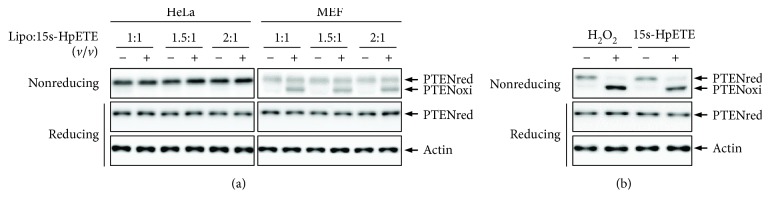
Effects of 15s-HpETE on the redox state of PTEN in HeLa and MEF cells. (a) HeLa and MEF cells were treated with different ratios (*v*/*v*) of 10 *μ*M 15s-HpETE and Lipofectamine 2000 transfection reagent for 5 min. (b) MEFs were incubated with 1 mM H_2_O_2_ or the mixture of 10 *μ*M 15s-HpETE and Lipofectamine (1 : 1 ratio) for 5 min. Cellular protein extracts were then alkylated with 10 mM NEM and subjected to nonreducing or reducing SDS-PAGE, followed by Western blot analysis using antibodies to PTEN or actin. “-” indicates Lipofectamine 2000 transfection reagent only; “+” indicates the mixture of 15s-HpETE and Lipofectamine 2000 transfection reagent or H_2_O_2_.

We further used Lipofectamine transfection reagent to assist 15s-HpETE penetration in cell membranes. HeLa cells were treated with different ratios (*v*/*v*) of 10 *μ*M 15s-HpETE and Lipofectamine reagent for 5 min, followed by Western blotting using the PTEN antibody. The data presented in Figure 3(a) shows that PTEN was rarely oxidized in the HeLa cells when treated with the mixture of 15s-HpETE and Lipofectamine reagent. We next tested the effects of 15s-HpETE on PTEN oxidation in MEF cells. As shown in Figure 3(a), more than 60% of PTEN was apparently oxidized, irrespective of the different ratios of 15s-HpETE and Lipofectamine reagent used. The observed changes in PTEN oxidation might come from injurious action of 15s-HpETE to cultured MEFs; therefore, we determined whether cell death occurred during treatment with the mixture of 10 *μ*M 15s-HpETE and Lipofectamine reagent. The cytotoxic assay results revealed that 10 *μ*M 15s-HpETE had a negligible effect on cell viability (data not shown). Therefore, a 1 : 1 *v*/*v* ratio of 10 *μ*M 15s-HpETE and Lipofectamine reagent was used in further experiments. It is well established that PTEN is predominantly modified into the oxidized form, with an intramolecular disulfide bridge between Cys^124^ and Cys^71^ residues, upon treatment with H_2_O_2_. To substantiate whether an identical intramolecular disulfide bond of PTEN was formed after 15s-HpETE treatment, lysates from MEFs treated with H_2_O_2_ or the mixture of 15s-HpETE and Lipofectamine were fractionated on nonreducing gels and probed with PTEN antibody. Faster migrating bands, similar to those seen in our previous studies [30, 34, 36, 39], were detected following H_2_O_2_ and a mixture of 15s-HpETE and Lipofectamine treatment (Figure 3(b)).

**Figure 4:**
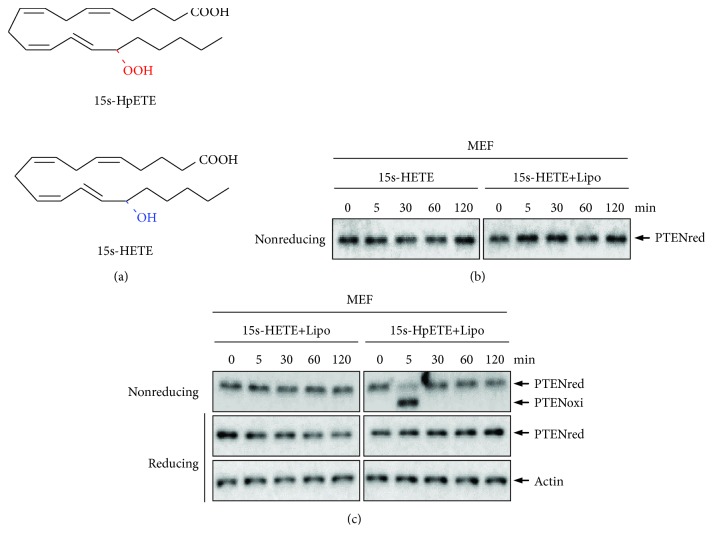
Effects of 15s-HETE and 15s-HpETE on the redox state of PTEN in MEF cells. (a) Chemical structures of 15s-HpETE and 15s-HETE. (b) MEF cells were treated with 10 *μ*M 15s-HETE or the mixture of 10 *μ*M 15s-HETE and Lipofectamine 2000 transfection reagent for the indicated times. (c) MEF cells were treated with the mixture of 10 *μ*M 15s-HETE and Lipofectamine 2000 transfection reagent or the mixture of 10 *μ*M 15s-HpETE and Lipofectamine 2000 transfection reagent for the indicated times. Cellular protein extracts were then alkylated with 10 mM NEM and subjected to nonreducing or reducing SDS-PAGE, followed by Western blot analysis using antibodies to PTEN or actin.

**Figure 5:**
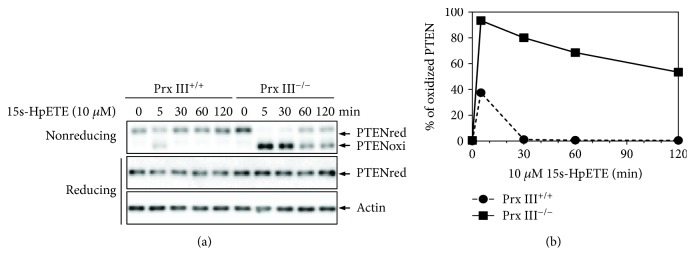
Effects of 15s-HpETE on the redox state of PTEN in Prx III^+/+^ and Prx III^−/−^ MEF cells. (a) MEF cells were treated with the mixture of 10 *μ*M 15s-HpETE and Lipofectamine 2000 transfection reagent for the indicated times. Cellular protein extracts were alkylated with 10 mM NEM and subjected to nonreducing or reducing SDS-PAGE, followed by Western blot analysis using antibodies to PTEN or actin. (b) The intensity of oxidized PTEN was quantitated using ImageJ software.

15s-HpETE is short lived in cells and is rapidly reduced to 15s-HETE by glutathione peroxidase 4 (GPx 4) [43, 44]. Previous reports have suggested that exogenous organic peroxides and hydroperoxides caused irreversible oxidation of PTEN [34, 36]. We, therefore, sought to characterize whether endogenous eicosanoids 15s-HpETE and 15s-HETE exert the same effects on the redox regulation of PTEN. As was to be expected showing in Figure 4(b), exposure of MEF cells with 15s-HETE or to the mixture of 15s-HETE and Lipofectamine reagent was unable to induce PTEN oxidation. MEF cells were then treated with the mixture of 15s-HETE and Lipofectamine reagent or the mixture of 15s-HpETE and Lipofectamine reagent for the indicated time points (0, 5, 30, 60, and 120 min). As depicted in Figure 4(c), PTEN was oxidized by 15s-HpETE in MEFs at 5 min after treatment and the oxidized PTEN was completely converted to the reduced form by cellular antioxidants after 30 min of treatment. Peroxiredoxins (Prx) are a superfamily of small nonseleno peroxidases that catalyze the reduction of H_2_O_2_, organic hydroperoxides, and peroxynitrite. Prxs play critical roles in protecting cellular components from oxidative damage [45]. Treatment of Prx III^−/−^ MEFs with 15s-HpETE enhanced PTEN oxidation at a higher level compared to that in Prx III^+/+^ MEFs (Figures 5(a) and 5(b)). In Prx III^−/−^ MEFs, approximately 90% of the PTEN was oxidized by 15s-HpETE at 5 min after incubation and the band intensity of oxidized PTEN decreased when the incubation time was extended, indicating that the oxidized PTEN was reduced by cellular antioxidants.

### 3.3. *15*s*-HpETE Inhibits Reduction of Oxidized PTEN by Inducing Trx Dimerization*

**Figure 6:**
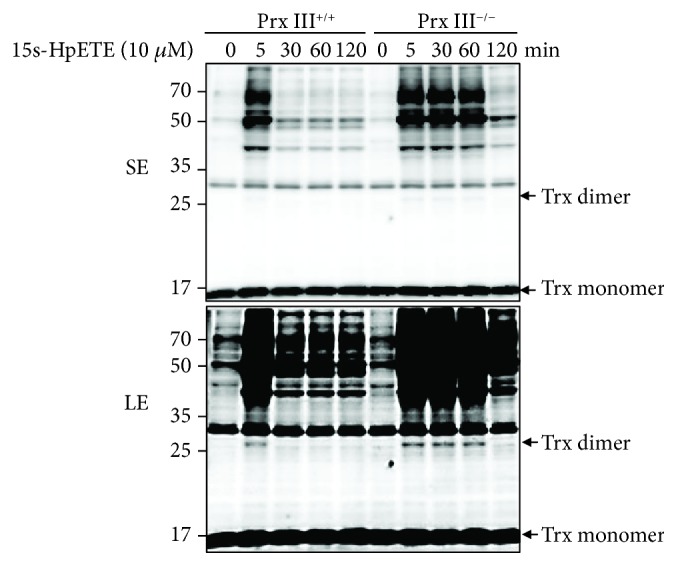
Effects of 15s-HpETE on the mobility of thioredoxin in Prx III^+/+^ and Prx III^−/−^ MEF cells. MEF cells were treated with the mixture of 10 *μ*M 15s-HpETE and Lipofectamine 2000 transfection reagent for the indicated times. Cellular protein extracts were alkylated with 10 mM NEM and subjected to nonreducing SDS-PAGE, followed by Western blot analysis using an antibody to thioredoxin. Trx: thioredoxin; SE: shorter exposure; LE: longer exposure.

Our previous studies revealed that oxidized PTEN induced by H_2_O_2_ was reversibly converted back to the reduced form by intracellular-reducing systems, predominantly by the Trx system. To test whether Trx was involved in the PTEN oxidation induced by 15s-HpETE, MEF cells were treated with the mixture of 10 *μ*M 15s-HpETE and Lipofectamine for the indicated times and the Trx status was analyzed using nonreducing SDS-PAGE (Figure 6). Trx dimers started to accumulate in Prx III^+/+^ MEFs after 5 min of incubation with 15s-HpETE. The dimeric forms protein completely converted to the monomeric forms after 30 min of incubation, which was reminiscent of the reduction kinetics of 15s-HpETE-oxidized PTEN. When Prx III^−/−^ MEFs were challenged with 15s-HpETE, dimerization of Trx was evident within 60 min; then the dimerization converted to the monomeric form after 120 min of treatment. In addition, high-molecular weight proteins and its dimer cooccurred after incubation with 15s-HpETE. These high-molecular weight bands may represent the oligomeric forms of Trx. It has been reported that Trx is functional as a monomer in redox reactions [46]. The accumulation of dimeric and high-molecular weight protein bands after incubation with 15s-HpETE could be attributed to impairment of the Trx-reducing system.

Based on these results, it could be concluded that endogenous lipid peroxide 15s-HpETE could inhibit tumor suppressor PTEN by reversible oxidation and simultaneous decrease of Trx activity by dimerization. In addition, Prx III played a critical role in protecting PTEN from lipid peroxide-induced oxidative inactivation.

## 4. Discussion

Our results demonstrated a previously unrecognized ability of endogenous lipid peroxides in the redox regulation of tumor suppressor PTEN. Lipoxygenases can catalyze the production of HpETE from arachidonic acid. These HpETEs are subsequently reduced and transformed to produce eicosanoids, important signaling molecules in immune responses and other physiological processes. A recent study reported that active lipid hydroperoxides formed by lipoxygenases can lead to diseases through cellular oxidative damage [47]. Oxidative stress and carcinogenesis are closely related; however, their specific molecular targets, especially the underlying mechanism involved in the promotion of prooncogenic signaling pathways, are scarce. PTEN, a member of the protein tyrosine phosphatase (PTP) family, is involved in the regulation of various cellular processes. PTEN deficiency is a hallmark of a variety of human tumors [19, 48]. It is accompanied by increased cell proliferation, decreased cell apoptosis, and enhanced Akt activity. The PTEN redox status is intimately linked to its enzymatic activity. Reversible oxidation of its catalytic Cys^124^ after hydrogen peroxide treatment can lead to the formation of a disulfide bond with Cys^71^ and inactivation of PTEN's phosphatase activity. Exposure of recombinant PTEN to 15s-HpETE resulted in increases of faster migrating bands in nonreducing SDS-PAGE, and such effects were completely reversed by treatment with DTT. 15s-HpETE-mediated cellular PTEN oxidation was identical to oxidation by H_2_O_2_, suggesting that an identical intramolecular disulfide bond was formed after 15s-HpETE treatment. 15s-HpETE was unable to induce cellular PTEN oxidation in C2C12, HeLa, or HT22 cells. This might be due to the fact that 15s-HpETE cannot cross phospholipid bilayers or that it was scavenged by cellular antioxidants. Even with the assistance of Lipofectamine, oxidized PTEN was not observed after 5 min of 15s-HpETE treatment in HeLa cells. This might be attributable to abnormal lipid metabolism in the transformed cells and warrants further study. Treatment of MEF cells with the premixture of 15s-HpETE and Lipofectamine induced oxidation of endogenous PTEN, further suggesting that 15s-HpETE could specifically mediate PTEN-related cellular responses. It is noteworthy that the concentration of 15s-HpETE (10 *μ*M) used to achieve substantial cellular PTEN oxidation was much higher than the physiological range [49]. Further studies should explore the intracellular levels of 15s-HpETE after treatment with a mixture of 15s-HpETE and Lipofectamine.

Prxs play key functions in the control of H_2_O_2_, organic hydroperoxides, and peroxynitrite reduction. Specifically, mitochondrial Prx III showed significant inhibition of PTEN oxidation induced by 15s-HpETE, suggesting that Prx III protected cells from oxidative damages induced by lipid peroxide. Human 15-LOX has two isoforms, 15-LOX-1 and 15-LOX-2. 15-LOX-1 is a dual-specificity enzyme that metabolizes AA principally to 15-HpETE and to far smaller amounts of 12-HpETE. 15-LOX-2 metabolizes AA to 15-HpETE and has little or no ability to metabolize AA to 12-HpETE. Mouse Alox15 metabolizes AA predominantly to 12-HpETE. We also investigated the effect of 12s-HpETE on the redox regulation of PTEN using MEF cells. 12s-HpETE showed a similar capacity of 15s-HpETE to oxidize and inactivate PTEN in MEFs (data not shown). Approximately 80% of PTEN was oxidized after 5 min of treatment with the mixture of 10 *μ*M 12s-HpETE and Lipofectamine, and the oxidized protein was converted to the reduced form within 30 min. Prx III deficiency enhanced 12s-HpETE-induced PTEN oxidation, and the oxidized PTEN was completely reduced after 60 min of incubation. However, the significance and mechanisms involved in 12/15s-HpETE-mediated PTEN oxidation need to be further elucidated.

Oxidized PTEN is converted back to the reduced form by cellular-reducing agents, predominantly by the Trx system. The Trx system, which consists of Trx, NADPH, and TrxR, maintains redox homeostasis in cells by catalyzing the conversion of protein disulfide to dithiol. The oxidative stress-mediated dimerization of Trx results in inactivation of the Trx system [35]. We previously reported that dimers and oligomers of Trx were increased after longer exposure times to organic peroxides and hydroperoxides, leading to the irreversible oxidation of PTEN [34, 36]. In the present study, Trx dimerization was not observed after 5 min of incubation upon exposure to 15s-HpETE in Prx III^+/+^ MEFs, consistent with PTEN oxidation. Trx dimerization was not observed after 60 min of incubation upon exposure to 15s-HpETE in Prx III^−/−^ MEFs. Oxidized PTEN was not reduced after 120 min of incubation in Prx III^−/−^ MEFs, indicating that Prx III might play a critical role in protecting PTEN, as well as the Trx system, from oxidation by lipid peroxide 15s-HpETE.

**Figure 7:**
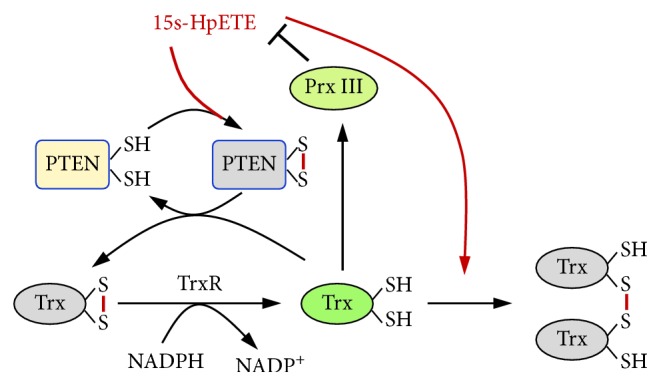
A schematic model of 15s-HpETE on the redox regulation of the tumor suppressor PTEN by the Trx system and Prx. Trx and Prx III play vital roles in the control of endogenous lipid peroxide-induced redox regulation of PTEN. 15s-HpETE inhibits the Trx redox system by inducing dimerization of Trx, resulting in the delayed reduction of oxidized PTEN and oxidized Prx. Prx III prevents 15s-HpETE-mediated PTEN oxidation by catalyzing the reduction of lipid peroxide. TrxR: thioredoxin reductase; Trx: thioredoxin; Prx: peroxiredoxin.

In this study, we showed that lipid peroxide 15s-HpETE could cause reversible oxidation of PTEN. Trx and Prx III were also involved in the 15s-HpETE-mediated redox regulation of PTEN (Figure 7). Taken together, our results unveil a new mechanism whereby lipid peroxides contribute to the etiology of tumorigenesis.

## Data Availability

The data used to support the findings of this study are available from the corresponding author upon reasonable request.

## References

[1] Brash A. R. (1999). Lipoxygenases: occurrence, functions, catalysis, and acquisition of substrate. *The Journal of Biological Chemistry*.

[2] Wisastra R., Dekker F. (2014). Inflammation, Cancer and Oxidative Lipoxygenase Activity are Intimately Linked. *Cancers*.

[3] Klil-Drori A. J., Ariel A. (2013). 15-Lipoxygenases in cancer: a double-edged sword?. *Prostaglandins & Other Lipid Mediators*.

[4] Kühn H., O’Donnell V. B. (2006). Inflammation and immune regulation by 12/15-lipoxygenases. *Progress in Lipid Research*.

[5] Dobrian A. D., Lieb D. C., Cole B. K., Taylor-Fishwick D. A., Chakrabarti S. K., Nadler J. L. (2011). Functional and pathological roles of the 12- and 15-lipoxygenases. *Progress in Lipid Research*.

[6] Kroschwald P., Kroschwald A., Ku¨hn H. (1989). Occurrence of the erythroid cell specific arachidonate 15-lipoxygenase in human reticulocytes. *Biochemical and Biophysical Research Communications*.

[7] Rothe T., Gruber F., Uderhardt S. (2015). 12/15-Lipoxygenase-mediated enzymatic lipid oxidation regulates DC maturation and function. *The Journal of Clinical Investigation*.

[8] Kim J. A., Gu J. L., Natarajan R., Berliner J. A., Nadler J. L. (1995). A Leukocyte Type of 12-Lipoxygenase Is Expressed in Human Vascular and Mononuclear Cells. *Arteriosclerosis, Thrombosis, and Vascular Biology*.

[9] Ochi H., Morita I., Murota S. (1992). Mechanism for endothelial cell injury induced by 15-hydroperoxyeicosatetraenoic acid, an arachidonate lipoxygenase product. *Biochimica et Biophysica Acta*.

[10] Middleton M. K., Zukas A. M., Rubinstein T. (2006). Identification of 12/15-lipoxygenase as a suppressor of myeloproliferative disease. *The Journal of Experimental Medicine*.

[11] Mahipal S. V. K., Subhashini J., Reddy M. C. (2007). Effect of 15-lipoxygenase metabolites, 15-(S)-HPETE and 15-(S)-HETE on chronic myelogenous leukemia cell line K-562: reactive oxygen species (ROS) mediate caspase-dependent apoptosis. *Biochemical Pharmacology*.

[12] Trachootham D., Alexandre J., Huang P. (2009). Targeting cancer cells by ROS-mediated mechanisms: a radical therapeutic approach?. *Nature Reviews. Drug Discovery*.

[13] Sultana C., Shen Y., Rattan V., Kalra V. K. (1996). Lipoxygenase metabolites induced expression of adhesion molecules and transendothelial migration of monocyte-like HL-60 cells is linked to protein kinase C activation. *Journal of Cellular Physiology*.

[14] Soumya S. J., Binu S., Helen A., Reddanna P., Sudhakaran P. R. (2014). 15-LOX metabolites and angiogenesis: angiostatic effect of 15(S)-HPETE involves induction of apoptosis in adipose endothelial cells. *PeerJ*.

[15] Rock C., Moos P. J. (2010). Selenoprotein P protects cells from lipid hydroperoxides generated by 15-LOX-1. *Prostaglandins, Leukotrienes, and Essential Fatty Acids*.

[16] Soumya S. J., Binu S., Helen A., Anil Kumar K., Reddanna P., Sudhakaran P. R. (2012). Effect of 15-lipoxygenase metabolites on angiogenesis: 15(S)-HPETE is angiostatic and 15(S)-HETE is angiogenic. *Inflammation Research*.

[17] Li J., Rao J., Liu Y. (2013). 15-Lipoxygenase Promotes Chronic Hypoxia–Induced Pulmonary Artery Inflammation via Positive Interaction With Nuclear Factor-*κ*B. *Arteriosclerosis, Thrombosis, and Vascular Biology*.

[18] Higgs G. A., Salmon J. A., Spayne J. A. (1981). The inflammatory effects of hydroperoxy and hydroxy acid products of arachidonate lipoxygenase in rabbit skin. *British Journal of Pharmacology*.

[19] Li J., Yen C., Liaw D. (1997). PTEN, a putative protein tyrosine phosphatase gene mutated in human brain, breast, and prostate cancer. *Science*.

[20] Di Cristofano A., Pandolfi P. P. (2000). The multiple roles of PTEN in tumor suppression. *Cell*.

[21] Finkel T. (1998). Oxygen radicals and signaling. *Current Opinion in Cell Biology*.

[22] Rhee S. G., Bae Y. S., Lee S. R., Kwon J. (2000). Hydrogen peroxide: a key messenger that modulates protein phosphorylation through cysteine oxidation. *Science Signaling*.

[23] Thannickal V. J., Fanburg B. L. (2000). Reactive oxygen species in cell signaling. *American Journal of Physiology. Lung Cellular and Molecular Physiology*.

[24] Rhee S. G. (1999). Redox signaling: hydrogen peroxide as intracellular messenger. *Experimental & Molecular Medicine*.

[25] Funamoto S., Meili R., Lee S., Parry L., Firtel R. A. (2002). Spatial and temporal regulation of 3-phosphoinositides by PI 3-kinase and PTEN mediates chemotaxis. *Cell*.

[26] Maehama T., Taylor G. S., Dixon J. E. (2001). PTEN and myotubularin: novel phosphoinositide phosphatases. *Annual Review of Biochemistry*.

[27] Luo J., Manning B. D., Cantley L. C. (2003). Targeting the PI3K-Akt pathway in human cancer. *Cancer Cell*.

[28] Stambolic V., Suzuki A., de la Pompa J. L. (1998). Negative regulation of PKB/Akt-dependent cell survival by the tumor suppressor PTEN. *Cell*.

[29] Kwon J., Lee S. R., Yang K. S. (2004). Reversible oxidation and inactivation of the tumor suppressor PTEN in cells stimulated with peptide growth factors. *Proceedings of the National Academy of Sciences of the United States of America*.

[30] Lee S. R., Yang K. S., Kwon J., Lee C., Jeong W., Rhee S. G. (2002). Reversible inactivation of the tumor suppressor PTEN by H2O2. *The Journal of Biological Chemistry*.

[31] Lee C. U., Hahne G., Hanske J. (2015). Redox modulation of PTEN phosphatase activity by hydrogen peroxide and bisperoxidovanadium complexes. *Angewandte Chemie (International Ed. in English)*.

[32] Schwertassek U., Haque A., Krishnan N. (2014). Reactivation of oxidized PTP1B and PTEN by thioredoxin 1. *The FEBS Journal*.

[33] Koharyova M., Kollarova M. (2015). Thioredoxin system - a novel therapeutic target. *General Physiology and Biophysics*.

[34] Han S. J., Zhang Y., Kim I. (2017). Redox regulation of the tumor suppressor PTEN by the thioredoxin system and cumene hydroperoxide. *Free Radical Biology & Medicine*.

[35] Watson W. H., Pohl J., Montfort W. R. (2003). Redox potential of human thioredoxin 1 and identification of a second dithiol/disulfide motif. *The Journal of Biological Chemistry*.

[36] Zhang Y., Han S. J., Park I. (2017). Redox Regulation of the Tumor Suppressor PTEN by Hydrogen Peroxide and Tert-Butyl Hydroperoxide. *International Journal of Molecular Sciences*.

[37] Conrad M., Sandin A., Forster H. (2010). 12/15-lipoxygenase-derived lipid peroxides control receptor tyrosine kinase signaling through oxidation of protein tyrosine phosphatases. *Proceedings of the National Academy of Sciences of the United States of America*.

[38] Covey T. M., Edes K., Fitzpatrick F. A. (2007). Akt activation by arachidonic acid metabolism occurs via oxidation and inactivation of PTEN tumor suppressor. *Oncogene*.

[39] Han S. J., Ahn Y., Park I. (2015). Assay of the redox state of the tumor suppressor PTEN by mobility shift. *Methods*.

[40] Lee S. R., Kim J. R., Kwon K. S. (1999). Molecular cloning and characterization of a mitochondrial selenocysteine-containing thioredoxin reductase from rat liver. *The Journal of Biological Chemistry*.

[41] Papa A., Wan L., Bonora M. (2014). Cancer-associated PTEN mutants act in a dominant-negative manner to suppress PTEN protein function. *Cell*.

[42] Heinrich F., Chakravarthy S., Nanda H. (2015). The PTEN tumor suppressor forms homodimers in solution. *Structure*.

[43] Cohen G., Riahi Y., Sasson S. (2011). Lipid peroxidation of poly-unsaturated fatty acids in normal and obese adipose tissues. *Archives of Physiology and Biochemistry*.

[44] Bryant R. W., Bailey J. M., Schewe T., Rapoport S. M. (1982). Positional specificity of a reticulocyte lipoxygenase. Conversion of arachidonic acid to 15-S-hydroperoxy-eicosatetraenoic acid. *The Journal of Biological Chemistry*.

[45] Rhee S. G. (2016). Overview on peroxiredoxin. *Molecules and Cells*.

[46] Weichsel A., Gasdaska J. R., Powis G., Montfort W. R. (1996). Crystal structures of reduced, oxidized, and mutated human thioredoxins: evidence for a regulatory homodimer. *Structure*.

[47] Spiteller G. (2003). Are lipid peroxidation processes induced by changes in the cell wall structure and how are these processes connected with diseases?. *Medical Hypotheses*.

[48] Bostrom J., Cobbers J. M., Wolter M. (1998). Mutation of the PTEN (MMAC1) tumor suppressor gene in a subset of glioblastomas but not in meningiomas with loss of chromosome arm 10q. *Cancer Research*.

[49] Coulon L., Calzada C., Moulin P., Vericel E., Lagarde M. (2003). Activation of p38 mitogen-activated protein kinase/cytosolic phospholipase A2 cascade in hydroperoxide-stressed platelets. *Free Radical Biology & Medicine*.

